# Different subtypes of motor cortex pyramidal tract neurons projects to red and pontine nuclei

**DOI:** 10.3389/fncel.2022.1073731

**Published:** 2022-12-20

**Authors:** Veronica Lopez-Virgen, Rafael Olivares-Moreno, Victor de Lafuente, Luis Concha, Gerardo Rojas-Piloni

**Affiliations:** Instituto de Neurobiología, Universidad Nacional Autónoma de México, Santiago de Querétaro, Mexico

**Keywords:** motor cortex, pyramidal tract neurons, sensorimotor cortex, corticorubral, corticopontine, layer 5

## Abstract

**Introduction:**

Pyramidal tract neurons (PTNs) are fundamental elements for motor control. However, it is largely unknown if PTNs are segregated into different subtypes with distinct characteristics.

**Methods:**

Using anatomical and electrophysiological tools, we analyzed in mice motor cortex PTNs projecting to red and pontine midbrain nuclei, which are important hubs connecting cerebral cortex and cerebellum playing a critical role in the regulation of movement.

**Results:**

We reveal that the vast majority of M1 neurons projecting to the red and pontine nuclei constitutes different populations. Corticopontine neurons have higher conduction velocities and morphologically, a most homogeneous dendritic and spine distributions along cortical layers.

**Discussion:**

The results indicate that cortical neurons projecting to the red and pontine nuclei constitute distinct anatomical and functional pathways which may contribute differently to sensorimotor integration.

## Introduction

Pyramidal tract neurons (PTNs) of the sensorimotor cortex are essential for motor control transmitting information directly to several subcortical structures including the spinal cord (Olivares-Moreno et al., 2017), medulla (Oswald et al., 2013), thalamus (Jiang et al., 2020), hypothalamus (Winnubst et al., 2019), and mesencephalic nuclei (Economo et al., 2018). PTNs are thick-tufted cells located in layer 5b of sensorimotor cortex that differ from intratelencephalic slender-tufted pyramidal neurons located in layer 5a, which project mainly to the striatum and contralateral cortex (Oberlaender et al., 2011). Despite PTNs having been extensively studied, it is largely unknown if they are segregated into different subtypes in the sensorimotor cortex.

Although several studies have shown that PTNs project almost individually to their respective targets (Akintunde and Buxton, 1992; Groh et al., 2010; Oswald et al., 2013; Rojas-Piloni et al., 2017), others have found that PTNs do not segregate into different projection types (Kita and Kita, 2012; Guo et al., 2017) with several collaterals of a single cell synapsing into more than one target. For example, PTNs projecting to subthalamic nucleus also send collaterals to the striatum, associative thalamic nuclei, superior colliculus, zona incerta, pontine nuclei (PN), multiple other brainstem areas and the spinal cord (Kita and Kita, 2012). Additionally, Guo et al. (2017) reported a high multidirectional projection index of L5 thick-tufted neurons, including corticospinal and corticostriatal neurons projecting to the midbrain, thalamus, and hypothalamus. This suggests that the sensorimotor cortex transmits parallel copies of information to several areas, but certain zones receive specific corticofugal information. In this way, recently (Economo et al., 2018) it has been found that corticothalamic and corticoreticular PTNs constitute distinct neuronal populations, with specific gene expression and electrophysiological activity related to tongue movement. However, neurons projecting to the midbrain, corticopontine and corticotectal nuclei seem to display fewer particularities (Rojas-Piloni et al., 2017; Economo et al., 2018).

Two important mesencephalic zones receive direct cortical inputs, the red nucleus (RN) and PN and both play a fundamental role in motor control, forming loops of information between the cortex and cerebellum in rodents (Liang et al., 2012; Wagner et al., 2019) and primates (Lemon, 2016). The parvocellular part of the RN receives ipsilateral projections from cortical neurons of sensorimotor areas (Humphrey and Rietz, 1976; Catman-Berrevoets et al., 1979), which in turn project to the ipsilateral lower olive and then to the cerebellum (Massion, 1967; Edwards, 1972; Walberg and Nordby, 1981; Kennedy, 1990). The magnocellular RN receives information from the nucleus interpositus of the contralateral cerebellum (Jansen and Jansen, 1955; Angaut and Bowsher, 1965; Courville, 1966; Daniel et al., 1988) and is the main origin of the rubrospinal tract (for a review, see Olivares-Moreno et al., 2021). On the other hand, PN receive ipsilateral inputs from several cortical areas in a topographical way (Schwarz and Thier, 1995; Huang et al., 2013), and then, the pontocerebellar tract reaches most parts of the cerebellum, constituting the major afferent input to the cerebellum (Brodal and Bjaalie, 1992; Brodal, 2014).

To understand the organization of PTNs in sensorimotor cortex here we analyzed anatomical, functional and morphological aspects of two classes of PTNs projecting to the RN and PN. The results indicate that, even though are topographically intermingled, distinct types of PTNs project to the RN and PN, suggesting that layer 5 output neurons are functionally compartmentalized.

## Materials and methods

### Animals

All procedures were carried out in strict accordance with the recommendations of the National Institutes of Health Guide for the Care and Use of Experimental Animals and the Laboratory Animal Care [Mexican Official Norm (NOM) 062-ZOO-1999]; the procedures were approved by the local Animal Research Committee of the Instituto de Neurobiología at Universidad Nacional Autonoma de Mexico (UNAM). We used 8-week-old male or female C57BL/6j mice that were maintained at constant room temperature (22 ± 2°C) under a 12-h light/dark cycle.

### Retrograde tracer injections

To quantify the number of corticorubral (CR) and corticopontine (CP) PTNs, we injected two retrograde neuronal tracers into the RN and PN. The animals were anesthetized with isoflurane/O2 gas (1.5%) and placed in a stereotaxic frame (World Precision Instruments, Inc., cat #E04008-005) and given an injection of 2% lidocaine (0.10 cc, s.c.) at the incision site. Then, a 1.5 cm incision across the midline was performed to expose the skull and identify the bregma suture. Small craniotomies were done with a dental drill over the injection sites using the following coordinates: RN 3.4 mm posterior from bregma, 0.7 from midline and 4 mm depth; PN 4 mm posterior from bregma, 0.5 mm from midline and 5.5 mm depth from the pia. Retrograde tracers were pressure injected (80–100 nL) using a picopump (WPI Inc., PV830 Pneumatic PicoPump) coupled to a calibrated glass injection capillary (BLAUBRAND^®^ intraMARK REF 7087 07). The volume delivered by capillary was supervised visually in each injection. After injection of tracers, the incision site was thoroughly cleaned with saline and sutured. Injections into RN and PN of the same animal were performed using a combination of the retrograde tracers Fluorogold (FG) (Fluorochrome, LLC; 3% in distilled water) and Dextran tetramethylrhodamine 3000 MW (BDA, Molecular Probes; 1 mg/ml in PBS, cat # D3308). Five days after the injections the mice were perfused transcardially with 0.1 M phosphate buffer (PB) followed by 4% paraformaldehyde in 0.1 M PB. The brains were extracted and post-fixed overnight in 50 ml of paraformaldehyde. Then the brain was cut coronally in the sensorimotor cortex at 50 μm in an automated vibrating Microtome (Leica VT1200S) and from the injection site. Slices in the injection site were mounted with SlowFade Gold (Molecular Probes, cat. num. S36937) after. The size of the injection sites was estimated automatically using ImageJ software (V 1.50i) outlining the periphery of the zone stained with the tracer in the center of each injection and computing the transversal area. Only the experiments in which the injections were located within the RN and PN were used for neuronal quantification and in which the injection sizes were equal. For NeuN staining, brain sections were rinsed three times in 0.1 M PB and incubated in a blocking solution for 40 min. Then, the sections were incubated in the primary antibody mouse anti-NeuN (dilution 1:500; Millipore, Cat # MAB377) in a blocking solution at 4°C overnight. Sections were rinsed 3x with 0.1 M PB and incubated in 0.1 M PB containing 10% goat serum and conjugated secondary antibody (Alexa Fluor 647 anti-mouse, dilution 1:500, Invitrogen cat #A-21235) for 1 h at room temperature, rinsed 3x and mounted with SlowFade Gold.

Mosaic images (resolution: 1.023 μm/pixel) of the sections containing the fluorescent retrograde-labeled cells were obtained on a fluorescence microscope (Zeiss AXIO Imager.Z1) attached to a digital camera (AxioCam MRm, 1.3 MP) using the appropriate filters (GFP for Alexa 488, Rhodamine for Alexa 594, and 650 nm for Alexa 647) and acquired with a 10x objective (ZEISS Plan-APOCHROMAT, NA: 0.45). Additional detailed images (resolution: 0.66 μm/pixel) were acquired with a confocal microscope (Zeiss 780 LSM) using an objective LD PCI Plan-Apochromat 25x/0.8 Imm Korr DIC M27.

### Analysis of the retrograde tracers

For the injection site quantification, we used ImageJ1.51u, and to analyze the projection neurons in the sensorimotor cortex the microscopy images (1.023 μm/pixel) were aligned manually in Amira (version 5.6) coupled to magnetic resonance image volume^1^ by selecting shared and clearly visible anatomical landmarks and using a linear transformation (Janke and Ullmann, 2015). Then when the microscopy images were aligned, the positions of the PTN somas were labeled. In this way, a 3D map of the CR and CP neurons was obtained. To compute relative neuron density, the soma distributions were obtained in 100 × 100 μm steps for the coronal plane, and vertical density profiles were computed in 50 μm steps along the vertical axes in M1.

### *In vivo* electrophysiological recordings and stimulation

For electrophysiological experiments 71 mice were used. Mice were anesthetized with isoflurane/O2 gas mixture (1.5%), placed in a stereotaxic frame, and maintained at a constant temperature (37°C). A small craniotomy was made in the coordinates for RN (3.4 mm posterior from bregma, 0.7 from midline and 4 mm depth; *n* = 34) or PN (4 mm posterior from bregma, 0.5 mm from midline and 5.5 mm depth from the pia; *n* = 37), and a bipolar concentric electrode (MicroProbes CEA 200) was positioned using a micromanipulator (Narishige SM-15L). Additionally, for the cortical recording of PTNs, a cranial window was made to expose M1 (0.98 mm posterior and 1 mm lateral relative to bregma). The *in vivo* juxtacellular recordings and biocytin fillings have been previously described in detail (Pinault, 1996; Narayanan et al., 2014). Briefly, recordings were performed with borosilicate glass pipettes (7–15 MΩ) and filled with normal rat Ringer’s solution with 2% biocytin (Sigma, cat # 576-19-2). The pipette was positioned with a micromanipulator (Luigs & Neumann, Mini Compact Unit). Electrophysiological signals were recorded in current-clamp mode using a microelectrode amplifier (Axon Instruments, Axoclamp 2B). Then, the output signal was amplified (x100) and filtered at a bandwidth of 10 Hz–10 kHz with a differential amplifier (AM systems, model 1700). The resultant electrophysiological signals were digitized with a rate of 20 kHz using Digidata interface (1440A) and the pClamp software module, Clampex (V 10.3).

The pipette was advanced in 1 μm steps to locate single neurons, indicated by an increase in the electrode resistance (25–35 MΩ) and in the amplitude of action potentials (3–5 mV). Ongoing spiking was recorded for each neuron during a minimum of 60 s. For each recorded cell we tested if antidromic spikes were evoked following RN or PN stimulation. Antidromic spikes were tested using a single, 0.1 ms square pulse with intensities starting at 100 μA and increasing until a threshold was reached for each cell, but never exceeding 300 μA. When a stable cell recording was obtained, the following criteria were used to establish the antidromic characteristic of the cell responses: a constant threshold and latency, the ability to follow a stimulus train of 333 Hz and a collision of the orthodromic spikes with antidromic evoked spikes (Olivares-Moreno et al., 2017). We used the spontaneous action potentials of sensorimotor cortex recorded neurons to trigger the electrical stimulation with a variable delay. Systematically changing the delay between the spontaneous spikes and stimulation allowed us to measure the critical period in which collision between spontaneous and evoked action potentials occurs. Neurons that did not satisfy these criteria were considered non-identified neurons.

Following the recording, juxtasomal biocytin filling was performed by applying continuous, low intensity square pulses of positive current (<7 nA, 200 ms on/200 ms off, Master-8, A.M.P.I.), while gradually increasing the current in steps of 0.1 nA and monitoring the action potential waveform and frequency. The membrane opening was indicated by a sudden increase in action potential frequency. Filling sessions were repeated several times (15–30 min) and diffusion was allowed for 1–2 h to obtain high-quality fillings.

After the experiments, the tip location of the stimulating electrodes was assessed with electrolytic lesions (100 mA, 10 s). At the end of the experiments, the animals were perfused, and coronal sections of interest regions were cut as indicated above. Slices with electrolytic lesions were mounted and stained with Nissl for electrode location analysis.

### Histology and image acquisition

Animals were perfused transcardially with 25 ml of 0.1 M PB solution followed by 25 ml of 4% paraformaldehyde in 0.1 M PB solution at pH 7.4. The brain was extracted and post-fixed overnight in paraformaldehyde. In experiments with *in vivo* recordings and biocytin filling, the cortex was cut into consecutive 50 μm thick tangential slices and treated with Streptavidin Alexa-488 conjugate (5 mg/ml Molecular Probes, cat num S11223) in PB with 0.3% TX for 3–4 h at room temperature to stain biocytin-labeled morphologies. All slices were mounted on glass slides, embedded with SlowFade Gold (Molecular probes, cat. num. S36937) and enclosed with a cover slip.

For the 3D reconstruction of individual dendritic morphologies (biocytin-488 nm), the images were acquired in a confocal system (Zeiss 780 LSM) with a x63 oil immersion objective (Zeiss, EC Plan-Neofluar 40x/1.30 Oil DIC M27) with a 488-excitation laser (emission detection range: 495-550 nm). Mosaic images were acquired at a resolution of 0.109 μm × 0.109 μm × 0.5 μm per voxel for ∼30 consecutive 50 μm thick brain slices to cover complete dendritic morphologies from the pia to L6.

### 3D PTN reconstruction and quantitative morphology

For the 3D tracing and reconstruction of the dendrite morphologies of PTNs (without knowledge of the PTNs’ subcortical targets), image stacks were deconvolved using ImageJ software for confocal images (ImageJ 1.50i, USA). Then, the FilamentEditor of Amira software (Amira V 5.6) with the semi-automated method of Oberlaender et al. (2007) were used and adapted for confocal microscopy (Rojas-Piloni et al., 2017). For the detection and automated reconstruction of dendritic spines, Neurolucida 360 software (Module AutoSpine 2.5 64-bits, Williston, Vermont, USA) was used.

Specific morphological characteristics of each reconstructed neuron were obtained (Table 2) and compared statistically for each group. The distribution of dendrite densities and dendritic spines every 50 μm from pia to L6 was statistically compared using the Kolmogorov-Smirnov test. Additionally, the similarity between these distributions was computed as follows: (1) each distribution of individual PTN was subtracted from each of the two target-related average distributions. If subtraction resulted in negative values, the respective bins were set to zero; (2) the bin-wise differences were summed across the entire depth (0–1,000 μm). This sum was defined as the similarity between the dendrite or spines (i.e., the smaller the similarity value, the more similar the distributions). The similarity values obtained from dendrites or spine density distributions for each PTN class were combined to two similarity indices, as shown in Figure 4: S.I. = $\frac{S\,i\,m\,i\,l\,a\,r\,i\,t\,y_{t\,o\,C\,P}-S\,i\,m\,i\,l\,a\,r\,i\,t\,y_{t\,o\,C\,R}}{S\,i\,m\,i\,l\,a\,r\,i\,t\,y_{t\,o\,C\,P}+S\,i\,m\,i\,l\,a\,r\,i\,t\,y_{t\,o\,C\,R}}$.

### Virtual tractography

To estimate the axonal length between M1 and either RN or PN, we queried the length of virtual tracts from the Allen Brain Connectivity Atlas (Oh et al., 2014). We selected 16 different axonal tracing experiments from the database with injection sites at M1 and targeting RN and PN (see list of tract tracing experiments below). The reconstructed streamlines were downloaded as json files through the streamline downloader service^2^ and converted to tck format using in-house routines built in Matlab R2018a. We combined all the streamlines from the 16 experiments into a single tractography file. Next, we used MRtrix3 (Tournier et al., 2019), a suite of tools built for diffusion-weighted MRI tractography to virtually dissect streamlines reaching either the ipsilateral RN or PN. The Allen Mouse Brain Atlas (Wang et al., 2020) was used to create binary masks of M1, RN and PN on the side of the injection site (Figure 3A). The resulting masks were used as inclusion criteria to dissect the tractography from M1, with three different criteria: [1] Streamlines reaching the ipsilateral PN (streamlines were truncated if they extended beyond PN, as they typically reached and arborized at the cerebellum); [2] streamlines reaching the RN by descending through the internal capsule then arching upwards to reach the ventral aspect of RN; and [3] streamlines reaching RN traversing the thalamus. Finally, we estimated the length of these pathways as the average distance of the streamlines included in each bundle.

### Tract tracing experiments from the Allen Mouse Brain Connectivity Atlas

180720175, 127084296, 100141780, 288169135, 166082842, 584903636, 126909424, 100141273, 277957908, 512130198, 298273313, 606250170, 272697944, 297711339, 156786234, 298325807, 100141563, 597007858, 168229113, 179641666, 166461193, 179640955, 182616478, 287461719, 177893658, 159651060, 310194040, 591612976, 157909001, 477836675.

### Experimental design and statistical analyses

Statistical analyses were computed using non-parametric tests. For multiple comparisons, a Kruskal-Wallis ANOVA and Friedman test were performed. To analyze the projection neuron profile in the motor cortex, and for the physiological data, we used the Shapiro-Wilk test to determine in every case if the data were normally distributed, and we applied the appropriate statistical test to establish statistical differences (one-way ANOVA or Kruskal-Wallis and Bonferroni or Tukey *post-hoc* tests, respectively) with Sigma Plot v12. To analyze the length and spines across the cortical depth we used a Kolmogorov-Smirnov test. The level of confidence was set at 95% (*p* < 0.05).

## Results

### Anatomical distribution of cortico-rubral and cortico-pontine neurons

**FIGURE 1:**
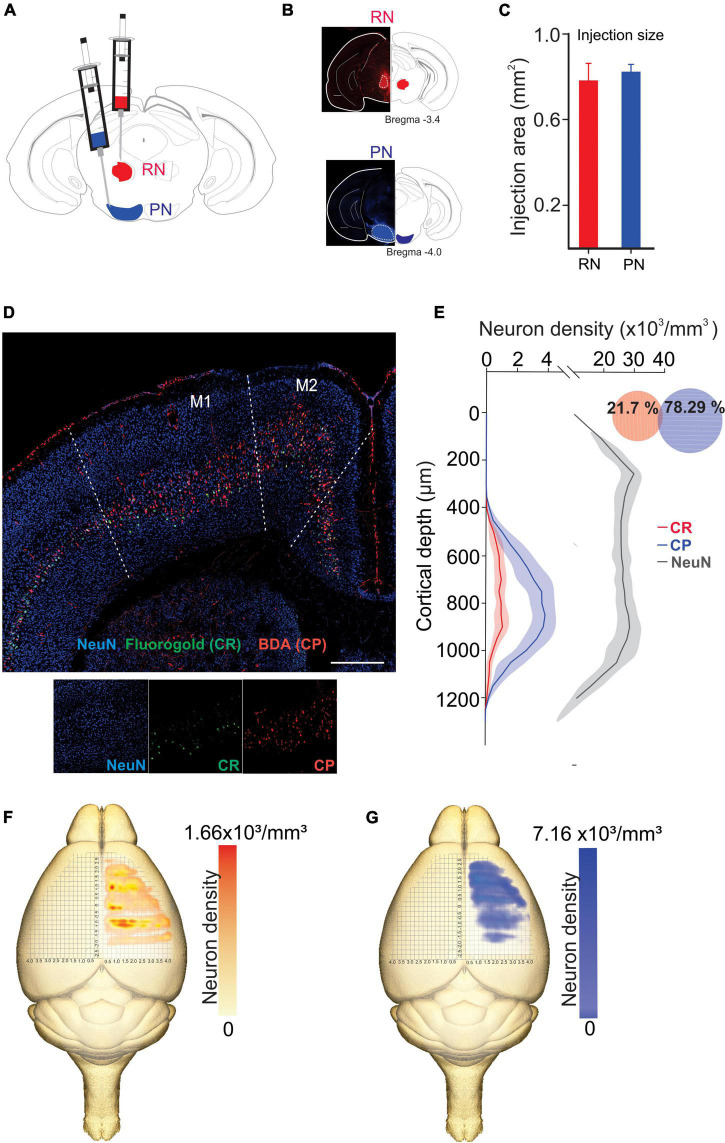
Distinct population of PTNs projecting to the RN and PN. **(A)** Retrograde tracers FG and ChT 594 were simultaneously injected into the RN and PN, respectively. **(B)** Examples of injection sites from the same animal. **(C)** Comparison of the injection extent, measured as the transversal area covered by the tracer [dashed lines in **(B)**], in 3 mice (RN = 0.7 ± 0.08 mm^2^, PN = 0.8 ± 0.03 mm^2^). **(D)** Coronal mosaic photomicrograph showing the distribution of CR (green) and CP (red) PTNs and NeuN (blue) in M1. Below detailed images of the 3 channels showing the cortical neurons. **(E)** Average vertical distributions of the CR (red) and CP (blue) PTN somata computed for 3 mice (3 consecutive slices per animal). Gray line represents the distribution of NeuN-labeled cells in the same experiments. Proportion of CR, CP, and double-labeled neurons (0.7 %) in M1 are indicated in the Venn diagram. **(F,G)** Averaged relative density representation maps (*n* = 3 animals) in the horizontal plane of the CR **(F)** and CP **(G)** neurons. Neuron density is expressed as the mean number of retrograde labeled neurons (neurons/voxel 250 mm^3^).

We analyzed the detailed distribution of CR and CP neurons in sensorimotor cortex. Two retrograde neuronal tracers were injected into the RN and PN (FluoroGold and BDA conjugated with rhodamine, respectively). Five days after injection, the animals were perfused for histological processing of the brain. The distribution of labeled cells was analyzed in 3 animals that were injected at both sites (Figures 1A–C, RN = 0.78 ± 0.081 mm^2^, PN = 0.82 ± 0.03 mm^2^, U = 97.00; *p* = 0.374, Mann-Whitney U statistic). Additionally, to obtain a 3D representation of the PTN density, the histological mosaic images were aligned with the MRI images of the mouse brain atlas (see text footnote 1; Ullmann et al., 2015). The results showed that both types of neurons are evenly distributed and intermingled in the rostro-caudal axis comprising the areas M1, M2, S1 and S2 (Figures 1D, F, G). However, most of labeled neurons are those corresponding to the cortico-pontine projection (∼80%; M1 F = 8.096, *p* = 0.010; M2 F = 11.173, *p* = 0.004; S1 F = 19.628, *p* = < 0.001; S2 F = 18.088, *p* = < 0.001, One way ANOVA, Table 1). Interestingly, the proportion of neurons projecting simultaneously to the RN and PN is less than 4% in all cortical areas (Table 1). To validate if both neuronal retrograde tracers can be used simultaneously for the analysis of the relative neuronal densities of projection neurons, we inject both tracers (FG and BDA) into the same area of the spinal cord. In this way, we observe a large proportion (81.3 ± 16.8, *n* = 3 experiments) of cells with BDA also are positive for FG, indicating that the existence of double-labeled cells could be due to neuronal co-termination.

**TABLE 1:** Percentage of CR and CP neurons in sensorimotor cortices.

	M1	M2	S1	S2
Corticorubral	18.9 ± 6.9	17.4 ± 6.7	15.5 ± 6.7	15.8 ± 7.3
Corticopontine	78.1 ± 8.1	78.4 ± 8.3	81.4 ± 8.3	81.4 ± 8.8
Double labeled	2.9 ± 1.6	4.1 ± 1.7	3 ± 1.5	2.7 ± 1.6

Previous results have found that some PTN showed differences in their depth distribution along layer 5 (Groh et al., 2010; Rojas-Piloni et al., 2017; Economo et al., 2018). However, here we found that specifically in M1, CR and CP neurons have essentially the same cortical depth distributions (Figure 1E) and there is not a significant difference in the depth below the pia in both groups (CP: 819.1 ± 8.517 μm, *n* = 393; CR: 789.4 ± 17.83 μm, *n* = 113; *p* = 0.1099, *t* = 1.602, Two-tailed student’s *t*-test). Moreover, a Kolmogorov–Smirnov test computed for the normalized distributions (*z*-score) of CR and CP neurons confirms that no differences exist (*p* = 0.9983) in the location of both cortical neurons.

### *In vivo* electrophysiological characteristics of cortico-rubral and cortico-pontine neurons

**FIGURE 2:**
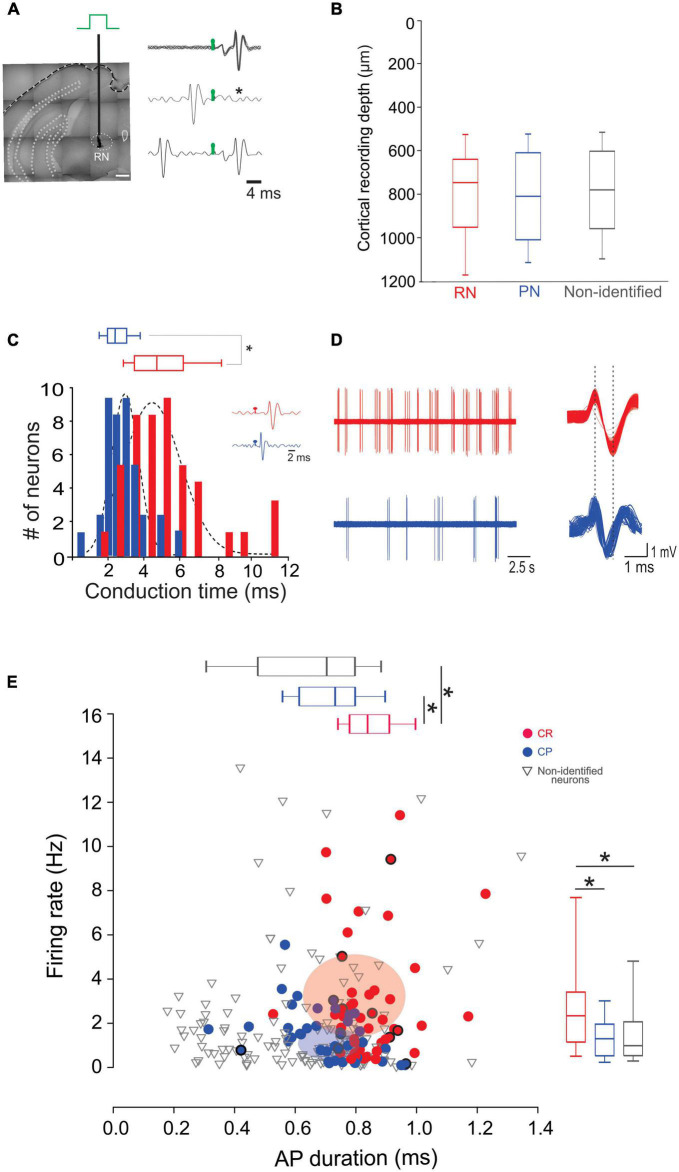
CR and CP PTNs exhibit distinct electrophysiological characteristics *in vivo*. **(A)** PTNs were identified by collision tests. A stimulating electrode was placed in the RN or PN to stimulate the CR or CP terminals. The representative traces show superimposed electrophysiological antidromic responses evoked by the ipsilateral RN (upper trace) showing that action potentials appear with a fixed latency (stimulation artifact in green). The middle trace shows the collision test when an orthodromic action potential occurs below the antidromic latency. The asterisk indicates the point at which the antidromic response would have occurred. The third trace shows that when the orthodromic action potential occurs above the antidromic latency, the collision of antidromic response does not occur. **(B)** Comparison between the recording depths obtained for PTNs projecting to RN, PN and non-identified neurons. **(C)** Distribution of antidromic conduction time of CR (red) and CP (blue) neurons showing that neurons projecting to the RN conduct significantly faster (*p < 0.05, Mann-Whitney). Two exemplary traces shown in the inset. **(D)** Representative electrophysiological recordings of spontaneous activity of CR (left upper trace) and CP (left lower trace). Aligned action potentials of the same neurons are also shown (right traces). **(E)** Relationship between action potential (AP) duration (peak to valley) and spontaneous firing rate of all recorded neurons (CR *n* = 45; CP *n* = 42; non-identified *n* = 154). Box plots show the distribution of AP durations (up) and firing rate (right). Ellipsoids in the graph represent the median (center) and standard errors (axis) of the data. *p < 0.05, One-way ANOVA, Dunn’s *post-hoc* test and Shapiro-Wilk test.

We next wondered whether neurons projecting to the RN and PN display distinct electrophysiological characteristics selective for their respective targets. For this, we performed single unit recordings of CR and CP neurons in M1, identified by the collision test of antidromic stimulation in the RN and PN (Figures 2A, B). Of the 242 neurons recorded, 45 projected to the RN, 39 to the PN, and 155 neurons had no identifiable projection to these nuclei. No differences were observed in the recording depth among CR, CP, and non-identified neurons (CR = 785.02 ± 32.1 μm, CP = 812.8 ± 33.9 μm, NI = 778.2 ± 19 μm; *F* = 0.373, *p* = 0.689, One way Analysis of Variance). However, CR neurons had a higher conduction time (5.09 ± 0.3 ms) as compared to CP neurons (2.54 ± 0.1 ms) (*U* = 1553.0, *p* < 0.001, Mann-Whitney U statistic) (Figure 2E). Moreover, the spontaneous firing rate of CR neurons was significantly higher than that of CP neurons (3.02 ± 0.41 Hz, 1.44 ± 0.17 Hz, respectively; *U* = 585.00, *p* = 0.002 Mann-Whitney rank sum test) and the duration of spontaneous action potentials of CP neurons was significantly lower than that of CR neurons (peak-to-valley duration; CR = 0.85 ± 0.01 ms; CP neurons = 0.72 ± 0.01 ms; *U* = 409.500, *p* = < 0.001, Mann-Whitney *U*-test, Figure 2E).

#### Axonal cortico-rubral and cortico-pontine projections display different conduction velocities

**FIGURE 3:**
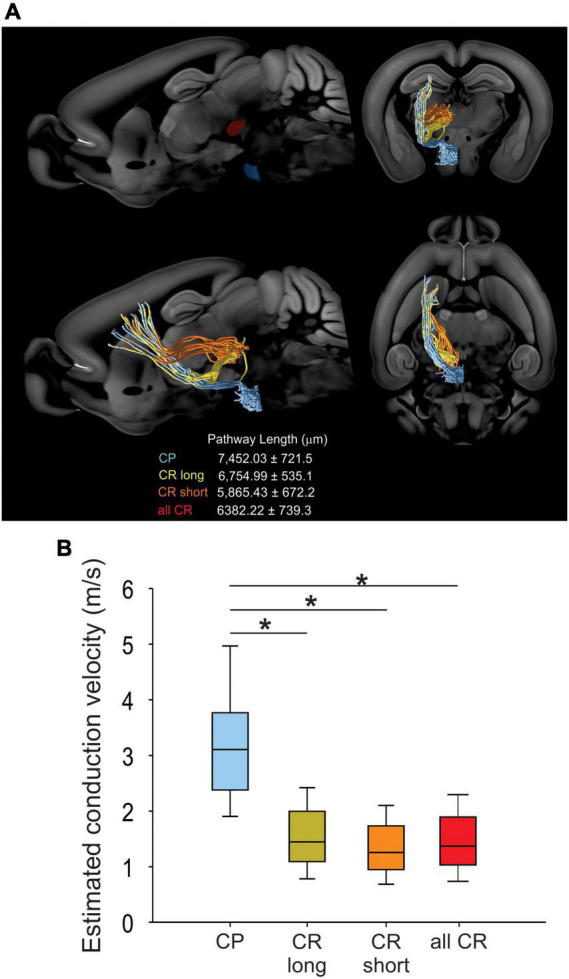
CR and CP neurons conduct at different velocities. **(A)** Visual tractography reconstructed from 30 axonal tracing experiments from the Allen Mouse Brain Connectivity Atlas, showing the target ROIs and the pathway length from CP and CR axons traveling through the thalamus (orange CR short) or substantia nigra (yellow CR long) in the different anatomical planes. **(B)** Comparison between the conduction velocities computed from conduction times (Figure 2C) and mean pathways lengths. Boxplots represent the median and 10–90 interquartile range. *p < 0.01 Kruskal-Wallis, One Way ANOVA, Dunn’s *post-hoc* test.

To analyze whether differences in antidromic conduction time are explained by differences in conduction velocity or merely a reflection of axonal length, we measured the length projections between M1 and RN or PN by means of virtual tractography (see “Materials and methods”). The length of the axonal projections between M1 and either the ipsilateral RN or PN were estimated as the average length of ipsilateral virtual tracts reconstructed from 30 axonal tracing experiments from the Allen Mouse Brain Connectivity Atlas (Oh et al., 2014). Descending axons travel together by internal capsule reaching the cerebral peduncle. As reported previously in the rat (Brown et al., 1977), CR axons leave the cerebral peduncle and penetrate the substantia nigra and medial lemniscus to enter the RN (CR long). However, some fibers exit the cerebral peduncle in a rostral manner and penetrate the thalamus to reach the RN (CR short) (Figure 3). The distance traveled by CP axons (7452.03 ± 721.5 μm, *n* = 2,305) is higher than that of CR bundles (CR long: 6754.99 ± 535.1 μm, *n* = 61; CR short: 5865.43 ± 672.2 μm, *n* = 44). With the measured mean pathway lengths of the tracts and the individual antidromic reaction times (Figure 2C), we computed the conduction velocities, which revealed that CP axons conduct significantly faster (3.64 ± 0.43 m/s) than CR axons (1.52 ± 0.11 m/s), without a difference between both pathways of CR neurons (*H* = 68.8, *p* < 0.001, Kruskal-Wallis One Way ANOVA; Figure 3).

### Morphology of cortico-rubral and cortico-pontine neurons

**FIGURE 4:**
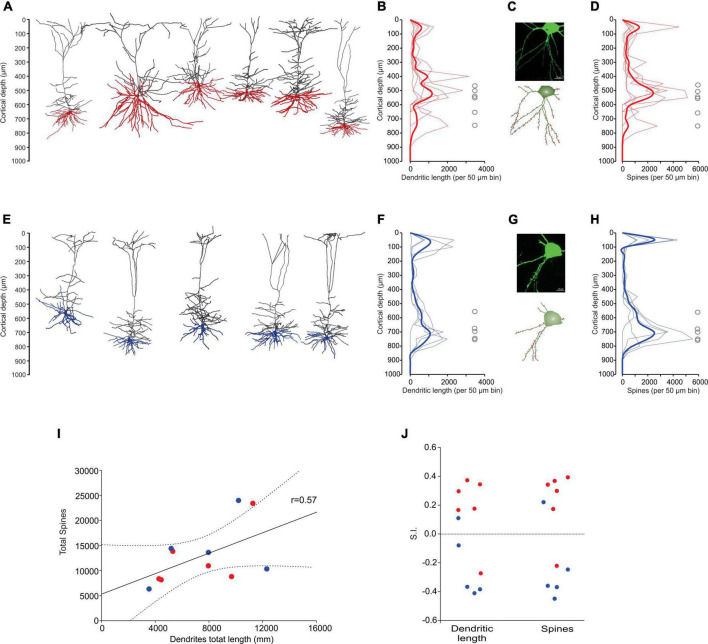
Dendrite morphology of PTNs reflects subcortical targets. **(A)** Three-dimensional reconstructions of labeled CR neurons identified *in vivo* (*n* = 6). Basal dendrites are indicated in red. **(B)** Distribution of dendrites along the vertical cortex axis. Each thin line corresponds to one neuron, and the average with the standard error is indicated by a thick line and shadow. The circles represent soma depth of each neuron. **(C)** Confocal microphotography of an exemplary CR neuron (upper) and its reconstruction (lower) showing the soma and some dendritic spines (red). **(D)** Distribution of dendritic spines along the vertical axis of the cortex. Each thin line corresponds to one neuron, and the average with the standard error is indicated by a thick line and shadow. **(E–H)** The same as **(A–D)**, but for CP neurons. **(I)** Dendritic path length per PTN is significantly correlated to the respective number of dendritic spines. (CR red circles, CP blue circles). Lines represent the linear regression and the upper and lower 99% confidence intervals. **(J)** Similarity indices (S.I.) between distributions along the cortex of dendrite density length and number of spines of each PTN. Notice that most of the similar neurons are of the same projection type.

Some of the *in vivo* recorded neurons were filled with biocytin by means of juxtacellular recordings and microiontophoresis, allowing us to reconstruct the dendrite morphologies of 11 labeled PTNs: 6 CR and 5 CP (Figures 4A, E). All reconstructed neurons had the same coordinates within M1 (0.98 mm posterior and 1 mm lateral relative to bregma). The tracing results were aligned to the pial surface, which allowed us to determine the 3D soma, dendrite locations and dendritic spines with 50 μm precision. The results show a different dendrite distribution between CR and CP neurons. The differences were quantified by comparing the distribution of the dendritic density (Figures 4B, F) and number of spines along the cortical depth (Figures 4D, H), which are different in CR and CP neurons (Dendrite density *h* = 1, *p* = < 0.0049; Dendritic spine number *h* = 1, *p* = < 0.001; Kolmogorov-Smirnov). Moreover, we quantified these differences by means of two indices to analyze the similarity (see “Materials and methods”) between dendrite and spine density distributions of each individual neuron and the two dendrite or spine averaged distributions across PTNs with the same target (Figure 4J). The similarity analysis revealed that both dendrite and spine density distributions along cortical depth were more similar when PTNs had the same target (dendritic length: CP Similarity to CP = 7167.4 ± 1183.2 vs. CP Similarity to CR = 11092.5 ± 669.2, *t* = −2.887, *p* = 0.02, CR Similarity to CR = 7972.9 ± 558.5 vs. CR Similarity to CP = 11957.1 ± 1600.3, *t* = −2.351, *p* = 0.041; dendritic spine: CP Similarity to CP = 8060.4 ± 1166.2 vs. CP Similarity to CR = 14008.8 ± 2887.9, *t* = −1.91, *p* = 0.09, CR Similarity to CR = 9305.0 ± 1036.9 vs. CR Similarity to CP = 15288.1 ± 2494.1, *t* = −2.215, *p* = 0.051).

For each PTN, we extracted a set of 9 morphological parameters (Table 2); no statistical differences between CR and CP neurons were found in any of the parameters. Additionally, the soma depth distributions of the CR- and CP-reconstructed neurons were similar to the overall distribution of the neurons labeled with retrograde tracers in M1 (range: 400–1,100 μm, peak: 850 μm, Figure 2). Soma depth locations were not significantly different between reconstructed CR (*n* = 6; 600 ± 36.515 μm) and CP (*n* = 5; 700 ± 27.386 μm) neurons (*t* = −2.113 *p* = 0.064, Student’s *t*-test).

**TABLE 2:** Total characteristics of the projection neurons to red nucleus and pons.

Dendritic features	Corticorubral neurons (*n* = 6)	Corticopontine neurons (*n* = 5)	*P*-value
**Total dendrites**	**Cell structure (mean ± SD)**		
Total dendrites length (μm)	7157.16 ± 1197.28	7839.90 ± 1598.56	0.735
Total nodes	876.83 ± 80.85	1083.6 ± 162.09	0.298
Intermediate nodes	643.83 ± 66.12	846.4 ± 142.21	0.792
Branching nodes	111.16 ± 8.57	113.4 ± 12.25	0.891
Terminal nodes	121.83 ± 9.54	123.8 ± 13.62	0.914
Segments number	796.16 ± 46.05	1084 ± 162.17	0.125
BoundingBox (mm^3^)	0.05 ± 0.01	0.07 ± 0.01	0.102
Spine number	12229.83 ± 2397.03	13712.00 ± 2931.97	0.702
Soma depth (μm)	574.8 ± 43.7	679.9 ± 35.1	0.103
	**Apical dendrites**		
Total dendrites length (μm)	5218.71 ± 1202.98	5610.70 ± 1272.61	0.828
Total nodes	437.66 ± 57.59	638 ± 112.5	0.160
Intermediate nodes	322.66 ± 43.97	499.4 ± 92.1	0.128
Branching nodes	46.83 ± 9.80	64.8 ± 11.71	0.303
Terminal nodes	61.5 ± 7.53	73.8 ± 11.87	0.427
Segments number	388.33 ± 37.60	636.6 ± 13.77	0.071
Spine number	8506.33 ± 1910.00	9126.40 ± 1907.82	0.662
	**Basal dendrites**		
Total dendrites length (μm)	1938.45 ± 344.40	2229.20 ± 458.78	0.618
Total nodes	439.16 ± 33.84	445.6 ± 58.16	0.929
Intermediate nodes	321.16 ± 25.91	347 ± 57.04	0.931
Branching nodes	58.33 ± 7.29	48.6 ± 1.67	0.298
Terminal nodes	59.66 ± 6.39	50 ± 2.07	0.250
Segments number	412.83 ± 27.59	447.4 ± 56.79	0.792
Spine number	3723.50 ± 819.10	4585.60 ± 1287.18	0.573
**Ongoing function (mean ± SD)**			
Firing rate (Hz)	3.59 ± 1.21	1.26 ± 0.45	0.185
Action potential duration (s)	0.87 ± 0.04	0.72 ± 0.08	0.125
Recording depth (μm)	697.33 ± 76.65	841.2 ± 43.87	0.082

## Discussion

We provided anatomical, electrophysiological and morphological evidence indicating that two distinct populations of PTNs project to the red and PN. Previous studies have described the relationships between structure and function in Layer 5 PTNs projecting to different targets (Groh et al., 2010; Rojas-Piloni et al., 2017; Olivares-Moreno et al., 2019). Our data revealed that both classes of PTNs are intermingled in sensorimotor cortices, including M1, and only a small proportion of neurons seem to contact the RN and PN simultaneously. This does not imply that PTN endings arrive only at a single subcortical area; for example, in the mouse barrel field it has been reported that around 40% of PTNs project to two targets, and 20% to three targets including the striatum, thalamus, hypothalamus, midbrain, pons and spinal cord (Guo et al., 2017). Moreover, Rojas-Piloni et al. (2017) reported that ∼80% of all PTNs in the barrel field project only to 4 targets (posterior medial division of the thalamus, tectum, pontine nucleus and subnucleus caudalis of the spinal trigeminal tract) and ∼20% of them project to two targets. Here, we confirm that the number of neurons projecting simultaneously to the RN and PN is considerably smaller (Akintunde and Buxton, 1992). However, it is to be expected that the majority of CR and CP PTNs innervate additional targets not explored in our study. The precise proportion of neurons projecting simultaneously to the red and PN remains to be determined because quantification of collateralization is always a crucial problem and usually, the double-labeled cells in multiple retrograde tracing experiments are underestimated (Vercelli et al., 2000).

Layer 5 thick-tufted PTNs are a canonical class of cells present in all neocortical areas (Ramaswamy and Markram, 2015) but also constitute the most morphologically and functionally variable type of neuron (Oberlaender et al., 2011; Kim et al., 2015; Rojas-Piloni et al., 2017). Here we found that CR and CP PTNs exhibit particular electrophysiological characteristics; that is, CR neurons have a higher firing rate and extracellular spike durations than CP neurons. This result confirms the previous findings showing that spike duration increases with increasing firing rate (Bourque and Renaud, 1985; Stratton et al., 2012). Both classes of neurons display different ongoing firing rates yet are in the same range of the previously reported PTNs in rats (Oberlaender et al., 2011; Rojas-Piloni et al., 2017).

In addition, we found that the antidromic spiking latency (conduction time) of CP neurons is shorter than in CR neurons, suggesting a higher conduction velocity for neurons projecting to the pons. Using virtual tractography we found two different pathways that CR neurons follow (CR short and CR long Figure 3). Surprisingly, the CR conduction times did not display a bimodal distribution, nevertheless, these neurons show a greater variability (broader distribution) of conduction time compared to CP. Additionally, CR short neurons have a slightly reduced estimated velocity compared to long (CR). However, because the conduction velocities were computed from conduction times, the conduction velocity of CR short neurons (Figure 3B) could be significantly underestimated.

We also demonstrated different conduction velocities for CR and CP neurons (1.5 and 3.6 m/s, respectively: range 1.3–18.6). The mean conduction velocities computed here are slightly less than conduction velocities reported in mice for the pyramidal tract (8.89 ± 1.81 m/s) (Tanaka et al., 2004), as well as for corticospinal tract (13.7 m/s) (Powers et al., 2012). Nevertheless, the discrepancies in conduction velocity estimated from anatomical or electrophysiological data have been explained by a recording bias (Towe and Harding, 1970; Kraskov et al., 2020) or by the precision to measure the distance traveled by the axons. The principal sampling bias rule for electrophysiological recordings is that the size of axon determines the probability of hit, but also neuronal shapes are involved (Towe and Harding, 1970). Here, we functionally identified particular classes of PTN neurons with different morphological characteristics. Thus, we can assume that previous reports have reported conduction velocities of the fastest PTN neurons.

By enriching our electrophysiological recordings with the extensive axonal tracing data set available through the Allen Brain Connectivity Atlas (Oh et al., 2014), we show a clear difference in conduction velocities between M1 neurons projecting to the RN and PN. The differences in conduction velocities might arise from different myelination properties of both classes of PTNs. In fact, a great variability in the longitudinal distribution of PTN myelin has been reported, suggesting the presence of a neuronal identity that could be used as a strategy to modulate long-distance communication in the neocortex (Tomassy et al., 2014). The main target of pontocerebellar projections are granule cells which have been considered as coincidence detectors (Wagner et al., 2019) because they receive inputs also from the brainstem and spinal cord (Sillitoe et al., 2012; Huang et al., 2013). Thus, timing in cortico-ponto-cerebelar pathway is important for sensorimotor integration. In this way, here we found that CP conduction times display less variability and a more homogeneous distribution between recorded neurons, implying that the information arrives with precise timing.

When we analyzed individual 3D morphologies of PTNs projecting to the RN and PN, no differences were found between global characteristics measured in both classes (Table 2). However, the distribution of dendrites and dendritic spines along the cortex is clearly different between CR and CP neurons, but similar between individuals of each class. Since layer-specific axon innervation is a feature of cortical organization, observed for local and long-range axons (Wimmer et al., 2010; Narayanan et al., 2015), the particularities in laminar distribution of target-related dendrites might suggest differences in the synaptic input that each PTN class receives. Moreover, these differences can produce distinct spiking patterns in both classes of PTNs. Due to the complex interaction between the dendritic and axonal initiation in different dendritic subdomains (apical vs. basal) of the same cell, the firing patterns of PTNs can be modulated by synchronous synaptic inputs arriving at different lamina (Larkum et al., 1999).

### Functional implications

Even though the role of the RN and PN in movement has been extensively described individually (Tziridis et al., 2009; Wagner et al., 2019; Basile et al., 2021), less is known about the specific contributions of each projection class. The function of the magnocellular part of the RN (that originates the rubrospinal tract) in movement has been well studied in behaviors like scratching, locomotion, and learned or automated motor behaviors (Kennedy, 1990; Gruber and Gould, 2010). However, there is little information about the parvocellular area that receives inputs from the cortex and originates rubro-olivary tracts. Lesions of the parvocellular region create mobility problems in rats, as it was shown that they could not perform precise movements, such as supination and pronation of the paw. They also exhibited deficiencies in digit movements (e.g., arpeggio) and had difficulty coordinating catching, aiming, and reaching efficiently (Whishaw et al., 1990, 1998; Morris et al., 2011, 2015). On the other hand, the PN also routes cerebral cortex information to the cerebellum (Brodal and Bjaalie, 1992; Wagner et al., 2019). PN unitary neuronal responses during forelimb movements in monkeys (Tziridis et al., 2009) have been recorded, and PN lesions in humans produce motor deficits (Schmahmann et al., 2004). A recent study tested the role of the PN in the execution of cortically dependent voluntary movements and indicated that it is essential for dexterous forelimb motor control (Guo et al., 2021).

Together, the electrophysiological and morphological results reinforce the idea that both classes of neurons process and conduct different information for sensorimotor integration and suggest that the sensorimotor cortex generates different downstream coding with separable contributions to volitive movements. Consequently, one of the most important goals for the next studies is to analyze the target-specific intracortical circuits that allow PTN to integrate specific features of sensory information to generate in parallel distinct descending commands in a coordinated manner.

## Data availability statement

The raw data supporting the conclusions of this article will be made available by the authors, without undue reservation.

## Ethics statement

The animal study was reviewed and approved by the Animal Research Committee of the Instituto de Neurobiología at Universidad Nacional Autónoma de México.

## Author contributions

GR-P conceived and designed the study and wrote the manuscript. VL-V carried out experiments. VL-V, RO-M, VL, LC, and GR-P performed the data analysis. All authors contributed to the article and approved the submitted version.

## Footnotes

1
http://imaging.org.au/AMBMC/Model

2
https://neuroinformatics.nl/HBP/allen-connectivity-viewer/streamline-downloader.html
